# Envisioning environmental equity: climate change, health, and racial justice

**DOI:** 10.1016/S0140-6736(23)00919-4

**Published:** 2023-07-01

**Authors:** Thilagawathi Abi Deivanayagam, Sonora English, Jason Hickel, Jon Bonifacio, Renzo R Guinto, Kyle X Hill, Mita Huq, Rita Issa, Hans Mulindwa, Heizal Patricia Nagginda, Priscila de Morais Sato, Sujitha Selvarajah, Chetna Sharma, Delan Devakumar

**Affiliations:** a Institute for Global Health, University College London, London, UK; b Lancaster Medical School, Faculty of Health and Medicine, Lancaster University, Lancaster, UK; c Institute for Environmental Science and Technology, Autonomous University of Barcelona, Barcelona, Spain; d International Inequalities Institute, London School of Economics and Political Science, London, UK; e Youth Advocates for Climate Action Philippines, Quezon City, Philippines; f Planetary and Global Health Program, St Luke's Medical Center College of Medicine—William H Quasha Memorial, Quezon City, Philippines; g Department of Indigenous Health, School of Medicine and Health Sciences, University of North Dakota, Grand Forks, ND, USA; h School of International Development, University of East Anglia, Norwich, UK; i Climate Operation, Kampala, Uganda; j School of Nutrition, Federal University of Bahia, Salvador, Brazil

## Abstract

Climate change has a broad range of health impacts and tackling climate change could be the greatest opportunity for improving global health this century. Yet conversations on climate change and health are often incomplete, giving little attention to structural discrimination and the need for racial justice. Racism kills, and climate change kills. Together, racism and climate change interact and have disproportionate effects on the lives of minoritised people both within countries and between the Global North and the Global South. This paper has three main aims. First, to survey the literature on the unequal health impacts of climate change due to racism, xenophobia, and discrimination through a scoping review. We found that racially minoritised groups, migrants, and Indigenous communities face a disproportionate burden of illness and mortality due to climate change in different contexts. Second, this paper aims to highlight inequalities in responsibility for climate change and the effects thereof. A geographical visualisation of responsibility for climate change and projected mortality and disease risk attributable to climate change per 100 000 people in 2050 was conducted. These maps visualise the disproportionate burden of illness and mortality due to climate change faced by the Global South. Our third aim is to highlight the pathways through which climate change, discrimination, and health interact in most affected areas. Case studies, testimony, and policy analysis drawn from multidisciplinary perspectives are presented throughout the paper to elucidate these pathways. The health community must urgently examine and repair the structural discrimination that drives the unequal impacts of climate change to achieve rapid and equitable action.

## Introduction

Climate change impacts the health of the planet and people; however, the impacts fall disproportionately on groups that are already disadvantaged. Racism, xenophobia, and discrimination are about division, control, and ultimately power, which are present in every society.^1^ Racism kills,^2^ and climate change kills.^3^ Racism, xenophobia, and discrimination interact with climate change to worsen existing harm to health and widen inequities for minoritised people both within and between the Global North and Global South^4^ (ie, individuals and populations who are denied equitable access to resources, social standing, and power; see the appendix pp 2–5 for a glossary of terms). This interaction is facilitated through institutionalised discriminatory policies and experiences of systemic oppressions by individuals and communities.^5^ In this Health Policy, we explore how several different forms of structural discrimination (based on caste, skin colour, ethnicity, race, Indigeneity, migratory status, and religion) interact with climate change and health. These distinct but intersecting vectors of inequality often result in poor health and underlying them are similar systems of categorisation, minoritisation, and oppression.^6^ The COVID-19 pandemic has highlighted the absence of global solidarity and willingness to redistribute resources to secure a safe route out of the pandemic. The same is true of climate change.

The most affected peoples and areas living in the Global South are often least responsible for climate change and yet bear its burden; but this also includes minoritised communities everywhere, such as Indigenous Peoples in the settler-colonial countries of the Global North (eg, the USA, Canada, and Australia).^7^ The majority of the responsibility for excess emissions lies with the states, corporations, and ruling classes of the Global North, in a manner reminiscent of the damages inflicted on people, land, and biodiversity during industrialisation and colonisation. In 2022, the Intergovernmental Panel on Climate Change explicitly identified “historical and ongoing patterns of inequity such as colonialism” as a factor in vulnerability to climate change.^8^ Global North–South inequality in responsibility and impact are intrinsically linked to discriminatory social and structural processes produced during colonialism. These processes continue today—eg, through the corporate destruction of land, excessive emissions, frequent exclusion of people from the Global South and Indigenous Peoples from international climate-related decision making, and placing the burden on minoritised people to develop less, slowly, or restrict their population to mitigate climate change.^9^, ^10^

This Health Policy has three aims. The first is to show the unequal health impacts of climate change due to racism, xenophobia, and discrimination, achieved through a scoping review of the literature. The second is to highlight unequal responsibility for climate change historically between countries, achieved through a geographical visualisation of secondary data, comparing responsibility for and the health burden of climate change with maps. Although Global North–South analysis can illustrate inequalities in responsibility and health burden between colonised countries and their ex-colonisers, and discrimination at the global level, they obscure inequities within countries. We also cannot infer causal links between unequal responsibility and unequal health burdens from solely Global North–South analysis. Thus, the third aim is to show the pathways through which climate change, health, and discrimination interact in the most affected areas across the world. These pathways are shown through case studies, testimonies, and policy analyses from multidisciplinary perspectives throughout the paper. This is a novel paper that presents the first academic review of literature on the interaction between climate change and discrimination leading to health inequalities, with quantitative geographical visualisations, qualitative case studies, and policy analysis to produce an encompassing analysis on this topic. We hope this can provide a robust platform for academics and practitioners to build discourse and justice-led action.

## Definitions

We define discrimination as unequal access to resources, political representation, and social treatment on the basis of caste, ethnicity, Indigeneity, migratory status, race, religion, or skin colour, unless otherwise specified.^6^ Further, per Crenshaw's notion of intersectionality,^11^ discrimination takes many forms and the lived experience of multiple forms of discrimination is greater than and distinct from the sum of each type of discrimination in isolation (see the appendix pp 2–4 for a glossary of terms). Above all, we stress that these categories assigned to people are socially constructed, born from colonial histories linked to ongoing separation and subjugation.

Other characteristics such as age, disability, gender, sexuality, and socioeconomic status (and the values placed on them) can exacerbate or mitigate experiences of discrimination, compounding the impact of climate change on health.^12^ Although this Health Policy focuses on the forms of discrimination specified previously, lived experiences are shaped by the relationship between overlapping systems of power and social categorisation.^13^ We acknowledge the commonalities in the role of coloniality, separation, and division in giving rise to discrimination based on social categorisations.^14^ Gender, disability, and income overlapping with racism are explored in this paper to understand interactions between social stratifiers.

There is no single definition of climate justice. Definitions range from Western conceptions (eg, Robinson's human-centred definition for activist spaces that insists on a movement in solidarity with people and communities most affected and sharing the impacts of climate change fairly^15^) to Global South conceptions (eg, the 2002 Bali Principles that demand ecological unity of all species). The Bali Principles insist that “communities have the right to be free from climate change, its related impacts and other forms of ecological destruction”.^16^ We acknowledge both definitions. We also recognise that climate justice must grapple with notions of climate debt and coloniality caused by centuries of ongoing oppression, felt deeply by generations of minoritised people,^17^, ^18^ and that populations already facing human rights violations also face the greatest human rights consequences of climate change.^19^ Environmental justice is also a relevant but distinct movement, which can largely be traced back to resistance to toxic waste dumping in poor, Black communities in the USA in the 1980s. This movement brought the civil rights, racial justice, and environmentalism movements together.^20^

## Methods

We used a multimethod approach to tackle our three central aims. The first section of the paper presents the findings of a scoping review on the health impacts of climate change, related to racism, xenophobia, and discrimination. The findings of the scoping review are presented according to the form of categorisation that they relate to. This section highlights health inequalities within countries.

The second section presents a geographical visualisation, highlighting inequalities in responsibility for climate change and the distribution of illness and mortality burden due to climate change between countries. In this section, national responsibility for climate breakdown is defined according to the fair-share approach.^21^ The fair-share approach rests on the principle that all people are entitled to use an equal share of the atmosphere within the safe planetary boundary of 350 parts per million concentration of carbon dioxide in the atmosphere.^22^ The maps visually represent the burden of illness and mortality attributable to climate change for climate-sensitive health outcomes in 2050.^23^ Indicators were chosen due to the availability of climate-related health data at the global level. Mortality and at-risk estimates were drawn from WHO,^23^ and population estimates from the UN World Population Prospects.^24^

The third aim of this paper is to show the pathways through which climate change, health, and discrimination interact. This is addressed throughout the paper with case studies, testimony, and policy analysis (presented in panels), as these methods are better suited than quantitative methods for analysing injustice in depth.^25^ These panels draw on perspectives from health workers, activists, youth (people aged 15–24 years), climate educators, anthropologists, and economists, integrating insights from community engagement activities, publicly accessible video testimony, and diverse bodies of collective knowledge from climate justice movement spaces with peer-reviewed literature. The topic of each panel was collaboratively decided by the authors and each panel was written or reviewed (or both) by individuals with localised expertise. The panels highlight the complex nature of the interactions between climate change, discrimination, and health, supplementing the overarching analysis presented in the scoping review and geographical visualisation sections.

## Climate change, health, and discrimination: a scoping review of the literature

This section identifies and analyses literature from a scoping review we conducted to identify the breadth of available evidence on the impact of climate change on health inequalities by race, ethnicity, caste, Indigeneity, religion, migratory status, and skin colour. The findings were limited to race, ethnicity, migratory status, and Indigeneity as no evidence was elicited in the search on caste, religion, or skin colour. Findings are summarised according to the form of categorisation that they relate to, with detailed findings in the appendix (pp 6–10).

**Figure 1:**
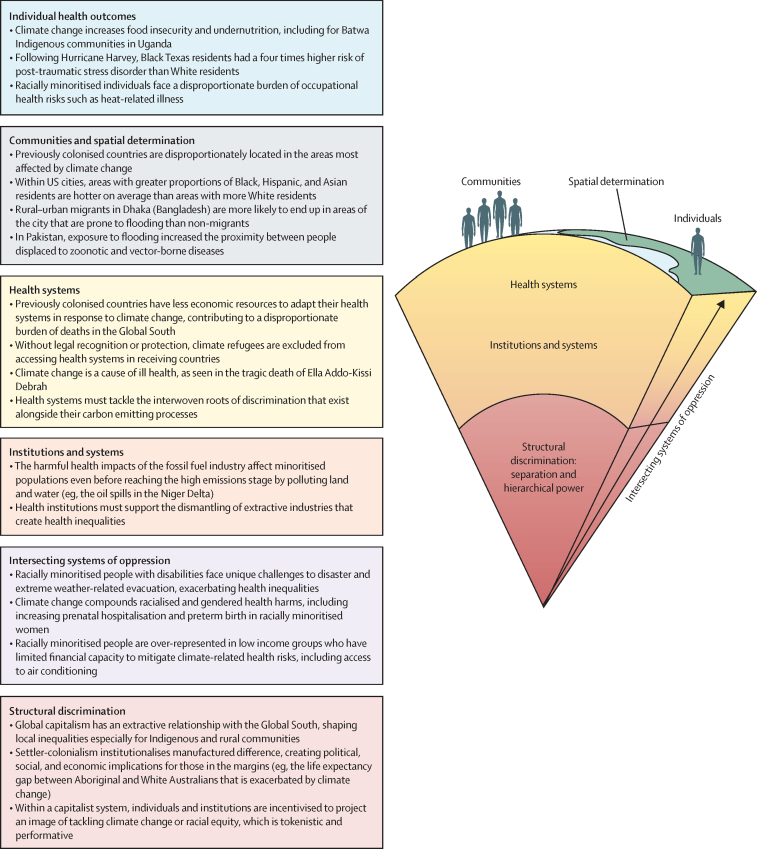
Devakumar and colleagues'^6^ conceptual model on racism, xenophobia, discrimination, and health applied to climate change and health Reproduced with permission of Devakumar and colleagues.^6^

Key themes from the scoping review are displayed in figure 1 with the use of Devakumar and colleagues' conceptual model from *The Lancet* Series on racism, xenophobia, discrimination, and health.^6^ This conceptual model highlights that health inequities are determined by active processes that occur within power structures, affect different levels of society, and impact biology across the life course. In figure 1, layers of society from the structural to individual level are represented by the layers from the core to the surface of the earth, illustrating the numerous dimensions of the interaction between climate change, health, and discrimination at each level. Specific examples are included throughout figure 1 to highlight how these interactions manifest across contexts and from the structural to individual level. A fully referenced version of figure 1 can be found in the appendix (pp 17–18).

### Race and ethnicity

17 studies from the USA and one from Canada addressed the relationship between climate change, health, and race or ethnicity (or both). Although the effects of race and ethnicity were searched separately, we summarise the findings together due to their overlap in the literature.

13 studies focused on heat exposure, heat sensitive illness, and mortality in the context of a warming climate. In the USA, census block groups (geographical units) with greater proportions of Black, Hispanic, and non-White residents have been found to be hotter on average than block groups with higher proportions of White residents within the same cities.^26^, ^27^ Areas with greater proportions of non-White residents also have heat-risk related land cover conditions (eg, little tree cover and high proportions of impervious surfaces), contributing to urban heat inequalities.^28^ Further, Black and Hispanic people are less likely to have air conditioning at home, after adjusting for household income, than White people.^29^, ^30^ In contrast, a study in Canada found no correlation between area level temperatures and proportion of visible minorities in Montreal, potentially due to rapid gentrification and lower levels of segregation than in the USA.^31^ The continued legacy of redlining, a policy of systematic disinvestment in minority and low-income neighbourhoods, could contribute to the inequitable distribution of heat exposure and heat-sensitive illness in the USA. Land surface temperatures and hospital admissions for heat-sensitive illnesses were higher in areas of cities previously targeted by this racist policy than areas not targeted.^32^, ^33^

Reflecting exposure patterns, heat-related asthma and acute myocardial infarctions are more common in Black and Hispanic people than White people in the USA.^29^, ^34^, ^35^ Further, hospital admissions for asthma, and cardiorespiratory and non-injury mortality, are more temperature-dependent in Black and Hispanic people than White people.^34^, ^35^, ^36^, ^37^ Climate-sensitive health risks can have intergenerational effects on racially minoritised families: Black women have a higher relative risk of preterm birth after exposure to heatwaves than White and Hispanic women, and exposure to extreme heat increases the risk of maternal hospitalisation more for Black women than White women.^38^, ^39^

Beyond heat-sensitive morbidity and mortality, racially minoritised groups are also more vulnerable to the physical and mental health impacts of extreme weather events and less likely to have their health-care needs met. For example, following the 2017 Hurricane Harvey, Black Texas residents were four times more likely to have high post-traumatic stress (scoring above 40 on the Post-Traumatic Stress Disorder Checklist–Specific).^40^ Racially and ethnically minoritised respondents were also more likely to report poor access to health care due to the hurricane than their White counterparts.^40^ Panel 1 presents a case study from Brazil on the interaction between racism against Quilombola people, climate change and deforestation, and their health. The case study highlights the complex and cascading risks linking discrimination, climate change, and health, showing relevant pathways not captured by the scoping review findings presented previously.

Panel 1 **Justice for Quilombolas in Brazil**

In Brazil, Quilombola communities, composed of descendants of enslaved African people with specific forms of communal social organisation, rely on the land and natural resources for their cultural, social, religious, ancestral, and economic reproduction. Under the Brazilian settler-colonial state, Quilombolas experience legal instability to their territorial rights, which (combined with poor access to health care, education, and other rights) reveals how historical disadvantages and institutional racism restricts the dignified survival of these populations.^41^ In several regions of the country, as in the Brazilian Amazon, land disputes of Quilombola and other traditional populations are intensified by mining and agribusiness. Brazil's previous governmental response reinforces threats to traditional communities and the Amazon rainforest, with the reduction of law enforcement and increased amnesty for deforesters.^42^ From August, 2020, to July, 2021, 13 235 km^2^ were deforested in the Brazilian Amazon region.^43^ A study in the region has shown that increases in deforestation combined with the intensification of the dry season worsens carbon emissions, risk of fire, and ecosystem stress.^44^

In addition to contributing to climate change, deforestation drastically impacts Quilombola communities. Residents of the Médio Itacuruçá community located in Abaetetuba, a city in the Brazilian Amazon, have reported the effects of weather unpredictability leading to crop failures and reduced food production in family farming. Older residents also reported that extreme heat has shortened the length of working days. These situations are aggravated by other forms of discrimination that leave Quilombolas more economically vulnerable to the delay in planting and the smaller harvests, creating susceptibility to food insecurity; almost half of Quilombolas are highly food insecure.^45^ Climate change threatens food security in communities around the world.^46^ Negative health outcomes associated with food insecurity include anaemia, asthma, cognitive problems, aggression and anxiety, depression, and suicide among children and poor mental health, diabetes, hypertension, hyperlipidaemia, and poor sleep among adults.^47^

The vulnerability of Quilombola communities to the health impacts of climate change is multidimensional, extending beyond the impacts of food insecurity while also aggravating it. Severe storms endanger housing of Quilombolas, as observed between December, 2021, and January, 2022, in several regions of Brazil. For example, in the state of Minas Gerais at least 72 communities across 20 municipalities were affected, leaving more than 7000 families in need of assistance, without food or water.^48^ The storms further hampered access to already marginalised communities, leaving populations without access to health care. Loss of biodiversity is a serious problem in the Amazon rainforest as it diminishes ecological niches occupied by predators of vector species and also creates new niches to alternative vectors, hosts, and pathogens. Moreover, deforestation and habitat loss are often accompanied by psychological aggressions (stress, malnutrition, and increased contact with pollutants), which can affect the immune system chronically and increase susceptibility to pathogens.^49^

Although protecting the environment is necessary for the existence of Quilombola communities, Quilombolas are also essential in ensuring the preservation of the environment and limiting the greenhouse effect. In Quilombola territories, nature is well conserved and highly valued, as their way of living centres around the rational and sustainable use of available natural resources. Therefore, assuring Quilombolas' rights and facilitating community leadership before measures that could affect the territory are necessary to achieving both historical reparation to the Quilombola population and reductions in greenhouse gas emissions.

### Migratory status

Ten studies from the USA (n=3), New Zealand (n=2), Australia (n=1), Austria (n=1), Bangladesh (n=1), Fiji (n=1), and Pakistan (n=1) highlighted the increased vulnerability of migrants to environmental hazards related to climate change. In Bangladesh, poor rural-to-urban migrants often migrate to low elevation coastal zones, increasing their risk from coastal flooding.^50^, ^51^ Internal migrants in Pakistan and Fiji face increased vulnerability to zoonotic diseases and non-communicable diseases due to dietary change, respectively.^52^, ^53^, ^54^, ^55^ In the USA, non-citizens are more likely to live in previously redlined areas where land surface temperatures are higher; are over-represented in flood-zones; and face greater exposure to harms associated with wildfires due to precarious work conditions and poor accessibility of disaster risk information.^32^, ^56^, ^57^ In Australia, migrant families reported higher perceived risk of heat-related health effects and decreased access to cooled public spaces.^50^ In addition to increased exposure to environmental hazards, people who have experienced international climate-induced migration face restricted access to health care, further increasing their vulnerability to climate-related health problems, highlighting the need for universal health care.^50^, ^52^, ^53^, ^58^

### Indigeneity

Eight studies discussed Indigenous communities in Uganda (n=3), the USA (n=2), Australia (n=1), Peru (n=1), and Tuvalu (n=1). Climate-related health concerns for Indigenous communities included malnutrition, malaria,^59^, ^60^ negative reproductive health outcomes,^61^ declining infant health,^60^ poor mental health,^62^ and respiratory distress.^63^ These outcomes were linked to colonialism's role in marginalising Indigenous communities, encroaching on land rights, and the impact that this has on their way of life.^64^, ^65^

In Batwa (Uganda) and Shawi (Peru) communities, malnutrition was driven by unusually extended rainy and dry seasons that negatively affected their primary food sources and added financial hardships that shaped food insecurity.^59^, ^64^ In Batwa communities, food insecurity was also linked to worse birth outcomes, such as smaller babies and a higher frequency of infant illness.^60^ In the same community, increased rainfall led to higher rates of malaria by increasing the amount of contaminated, stagnant water present in the community, whereas the dust of extended dry seasons led to respiratory problems.^59^ In Houma Nation (USA), women observed an increase in reproductive health concerns including eclampsia, gynaecological cancers, and preterm birth.^61^ Sociopolitical exclusion and socioeconomic hardship amplified the health impacts of climate change faced by Indigenous communities. For example, land insecurity restricts Batwa communities' socioeconomic mobility and low socioeconomic status restricts their access to household goods for infection prevention (eg, boiling contaminated water)^59^ and financial access to education.^64^

### Intersecting systems of oppression

An intersectional approach is necessary to understand and respond to health inequalities resulting from interacting power structures.^14^ Two intersecting systems of oppression are addressed in the findings of our scoping review: gender and race; and income and race. None of the papers reviewed presented findings on age or sexuality. Visually, intersectionality is represented in Devakumar and colleagues' model as an arrow cutting across various strata of society, representing how minoritisation and discrimination result from multiple systems of power operating within their own historical and structural contexts (figure 1).

#### Gender and race

Two studies focused on the environmental determinants of racial inequalities in maternal health; in addressing gendered health issues, they highlighted that women face additional climate-related health vulnerabilities and that racially minoritised women are doubly disadvantaged.^38^, ^39^ However, none of the studies applied intersectionality to analyse the role of gender in shaping inequalities faced by minoritised people or addressed the health burden faced by people of other marginalised genders (ie, other than cisgender women). Nevertheless, there is a large body of work on gendered climate-related health inequalities.^66^, ^67^ Panel 2 shows the interaction between colonialism, gender, and health in the Philippines and Uganda through a comparative policy analysis.

Panel 2 **Comparative policy analysis of gender mainstreaming in the climate–agriculture–health policy nexus in the Philippines and Uganda**

Gender mainstreaming has been integrated into climate policies in many countries, including the Philippines and Uganda, to address the health inequities faced by women agriculturalists due to a changing climate. Mainstreaming is a policy approach that integrates elements across distinct issue areas towards one policy agenda. Gender mainstreaming can redress structural inequalities that result from colonial legacies, including gender inequity. Such structural inequalities shape the uneven distribution of climate change's impact on health, causing women and minoritised groups to be disproportionately affected.

Under Spanish, American, and British occupation, the Philippines and Uganda experienced colonial histories that continue to diminish the role of women in society compared with pre-colonial times. For instance, in pre-colonial Philippine society, women held leadership roles in spiritual and scientific affairs;^68^ in Uganda, the value system introduced by colonialism devalued the role of women in society by depicting their work as traditional and backwards.^69^ Colonial structures still shape how both Filipino and Ugandan women experience health, economic, and social deprivations.

The nexus of agriculture, climate change, and health exemplifies the lasting impacts of colonially driven gender inequity. Climate change will devastate the health of small scale and subsistence farmers, particularly through reducing food security and nutrition. In both the Philippines and Uganda, these health implications will be felt more acutely by women. In Uganda, women constitute 76% of the agricultural labour force^70^ and are disproportionately represented in subsistence-oriented farming of low-value crops. Women are less represented in the Philippine agricultural labour force, but consistently have lower wage rates and purchasing power relative to men.^71^ The marginalisation of women increases their vulnerability to climate change-related agricultural shocks including drought, flooding, and crop failures. Women additionally face unique climate impacts on maternal, reproductive, and sexual health.^72^, ^73^ For instance, following the 2013 Typhoon Haiyan in the Philippines, pregnant women were more vulnerable to undernutrition and miscarriage and adolescent girls to sexual-based violence.^74^

The Philippine Magna Carta for Women^75^ protects women affected by disasters and calamities, empowers women farmers, and ensures access to healthy food for women and girls. Further, the Climate Change Act^76^ of 2009 and the People's Survival Fund Act^77^ of 2012 both promote gender mainstreaming in climate adaptation and disaster risk reduction. Meanwhile, the Ugandan National Climate Change Act^78^ 2021 includes valuable considerations of gender and food security, showing attention to the specific needs of women agriculturalists. Nevertheless, gender mainstreaming efforts have not substantially mitigated the risks faced by Filipino and Ugandan women. In Uganda, gender and food security are unsystematically integrated into climate and food security policies^79^ and in the Philippines, community-level implementation of climate disaster risk management policies are insufficient and devoid of gender considerations.^80^, ^69^ Addressing the implementation challenges of gender mainstreaming is key to ensuring effective and sustainable policy responses. Although gender mainstreaming might be frequently advocated by international organisations, the design and implementation of such policies must consider the idiosyncrasies of local contexts (including colonial histories) and incorporate grassroots, community-led interventions. In addition to embedding gender mainstreaming in domestic climate policy, it must be integrated across other sectoral policy areas, given the climate's overarching impact on society and government. Finally, gender mainstreaming must also incorporate a broader anti-colonial and anti-imperialist approach to address all forms of inequality—eg, racial discrimination, which oftentimes overlaps with gender inequality.

#### Income and race

Income and race are two axes of oppression that closely interact.^14^ Many of the papers (n=16) addressing race and ethnicity in our scoping review also highlighted the importance of income in mediating climate-related racial health inequalities. For example, the literature highlighted the disproportionate burden of extreme heat and heat-sensitive illnesses faced by people and areas with low-income,^27^, ^31^, ^34^ their poor access to cooling facilities and resources, and their exclusion from green infrastructure initiatives,^26^, ^29^, ^30^ demonstrating structural racism.^1^, ^81^ None of the studies examined how race or ethnicity intersect with socioeconomic status to compound health inequalities related to climate change. Further, the reviewed literature failed to highlight that poorer and marginalised people who experience the greatest impacts of climate change have contributed to it the least, as shown in a large body of work.^82^, ^83^, ^84^

### Summary

Overall, the findings indicate an unequal distribution of the health impacts of climate change, disproportionately burdening racially and ethnically minoritised groups, migrants, and Indigenous Peoples. These findings are supported by the Intergovernmental Panel on Climate Change, which has highlighted that people who face social marginalisation due to ethnicity and Indigeneity are among those most vulnerable to the impacts of climate change.^8^ The numerous dimensions of the interaction between climate change, health, and discrimination at each level of society are presented through application of Devakumar and colleagues' conceptual model in figure 1.^6^ The evidence is concentrated in high-income settings, but low-income settings are also represented. Although the evidence summarised here points towards an unequal distribution of health impacts of climate change, unequal responsibility for climate change was not highlighted in the body of literature reviewed here, suggesting a gap in research linking unequal responsibility with health effects. Yet, inequities in responsibility and burden are visible within and between countries. Discussion of unequal responsibility alongside the health inequalities identified in this scoping review is essential to identifying and dismantling the power structures at the root of climate-related health inequities. As such, the next section of this paper analyses inequalities in responsibility and health impacts of climate change through presentation of a geographical visualisation.

## Inequalities in responsibility and health impact: a geographical visualisation

### Unequal responsibility for climate change

There is a tendency to refer to climate change in the language of the so-called Anthropocene. This terminology is useful to the extent that it highlights the anthropogenic causes of the crisis; however, not all humans are equally responsible for the problem. Climate change is ultimately an effect of the economic system: capitalism, which seeks to cheapen labour and resource inputs, often through processes of extraction and exploitation, including through historical and ongoing processes of colonisation, dispossession, and coercion.^85^ Ideologies of race and other forms of categorisation have long been used to justify these processes (figure 1).^86^ Capitalist dynamics have produced severe global inequalities, with centres of wealth and capital accumulation characterised by high amounts of consumption and energy use, hindering the ability of some populations to meet their basic needs.

Centres of accumulation in the Global North are overwhelmingly responsible for greenhouse gas emissions that exceed the safe planetary boundary, whereas the Global South suffers the effects disproportionately. With the fair-share approach, described in the methods section, it is possible to determine the extent to which nations have exceeded their fair share of the safe emissions budget and thus how much they have contributed to pushing global emissions beyond the safe planetary boundary. Cumulative emissions exceeded this level in the late 1980s, driving the crisis of climate breakdown that is evident today.

**Figure 2:**
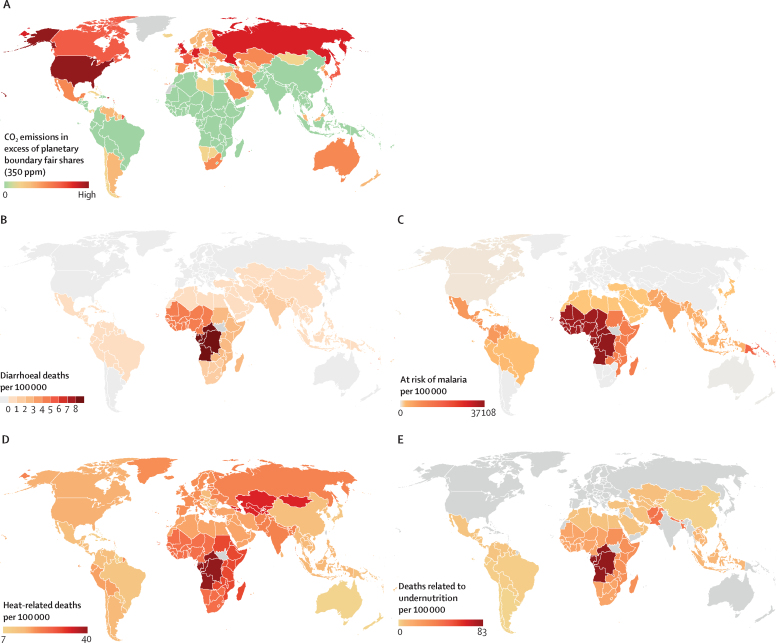
Unequal responsibility for climate breakdown and unequal health outcomes between countries (A) Responsibility for climate breakdown across countries based on cumulative CO^2^ emissions from 1850–2015; countries in green were still within their fair share of the 350 ppm boundary as of 2015. (B) Projected climate-change attributable diarrhoeal mortality in people aged younger than 15 years; countries in light grey have mortality less than 0·01. (C) Projected climate-change attributable population at risk of malaria; countries in light grey have a risk of 0, the USA and Canada have a risk of 27. (D) Projected climate-change attributable heat-related mortality in people older than 65 years. (E) Projected climate-change attributable mortality due to undernutrition in children younger than 5 years; grey indicates no data. Projected climate change-attributable mortality and disease risk maps were created using Microsoft Excel and Datawrapper software to visually display mortality rates and population at risk per 100 000. Rates were calculated with the estimated number of deaths and population at risk per region in 2050 and medium population growth estimates for 2050 from the World Population Prospects 2010 revision.^23^, ^24^ PPM=parts per million.

Assessing cumulative emissions over the period 1850–2015, countries of the Global North, which represent 14% of the world's population, are responsible for 92% of historical carbon dioxide emissions in excess of the safe planetary boundary.^21^ Most countries in the Global South (including large, populous countries like India, Indonesia, and Nigeria) are still well within their fair share of the planetary boundary (figure 2). This dynamic can be understood as a process of atmospheric colonisation.^87^ Just as land and territories have been colonised in the past, so too have the atmospheric commons been appropriated by wealthy countries for their own enrichment, through forms of industrialisation and growth that have relied on colonial patterns of appropriation, with devastating consequences for all life on Earth.^88^ In this way, climate change showcases colonialism as a historically rooted, yet ongoing structure, that governs and shapes our lives, “which are co-constitutive of processes of capitalism, imperialism, and international development”.^18^ This visualisation shows not only where economic, social, and political dominance lies, but also which economies are more responsible for repairing damage.

Not all people in the Global North are responsible for excessive emissions, just as not everyone in the Global South is less responsible. Here, class inequalities prevail; wealthy individuals are disproportionately responsible for excess emissions given their disproportionate influence over production and legislation, but also in terms of their personal consumption. The average carbon footprint of the top 1% of emitters is 75 times higher than the bottom 50% of emitters.^84^ Corporations bear a specific responsibility here, and have long exploited racial divisions—eg, through damaging the territories of minoritised groups, exemplified by the case study of Quilombolas. In all settings, groups who are minoritised experience the health impacts in their settings while consumption occurs in global centres of wealth.^89^, ^90^

### Health inequalities between countries

According to the Climate Vulnerability Monitor, of the estimated 400 000 deaths caused by climate change globally in 2010, 98% occurred within the Global South.^91^ Figure 2 displays the unequal global burden of mortality attributable to climate change from heat-related mortality, undernutrition, and diarrhoea, and the population at risk of malaria in 2050. The data used in each map accounts for base case economic growth and an emissions scenario consistent with A1b (see appendix p 19 for descriptions of emissions scenarios).^23^ The responsibility for the climate breakdown map^92^ is based on overshoot emissions beyond the 350 parts per million planetary boundary.^21^

Geographically uneven patterns of climate-related health impacts are apparent, with risk of exposure to deadly heat and heat-related mortality^93^, ^94^, ^95^, ^96^ and food insecurity^97^, ^98^ concentrated in the Global South. Multidimensional climate vulnerability, which assesses vulnerability indicators related to food, water, health, ecosystem services, human habitat, and infrastructure, is likewise higher in the Global South than the Global North.^99^

The vulnerability of Global South countries to climate change is due in part to the concentration of negative biophysical effects in tropical zones, but also to colonial histories and persistent structures, including trade liberalisation, predatory financing arrangements, and debt accrual by the International Monetary Fund and World Bank beginning in the 1980s.^100^, ^101^ Countries affected by these measures often do not have the capacity to respond and adapt to climate-related health impacts, and benefits of adaptation will thus be concentrated in countries with the economic power necessary to invest in adaptive technologies and behaviours.^94^ Panel 3 is a case study from the Marshall Islands, a nation that still remains well within its fair share of the planetary boundary but is highly vulnerable to climate-related health inequalities driven by racial injustice.

Panel 3 **Climate, health, and racial apathy in the Marshall Islands**

Despite being well within their fair share of the planetary boundary and facing geographical isolation from the financial benefits of a fossil fuel-driven global economy, large ocean states are uniquely vulnerable to climate change.^102^ The Marshall Islands is among the most climate-vulnerable countries in the world due to the risks associated with sea level rise, given its low altitude (averaging less than 2 metres above sea level), densely populated coastlines, and concentration of more than 95% of its infrastructure in the Low Elevation Coastal Zone.^8^, ^103^ Under a high emissions scenario, the Marshall Islands is projected to have annual wave-driven flooding over its entire surface by 2060–2070.^104^ For this atoll nation, climate change is a current reality already affecting the Marshallese and the survival of their nation.

Marshall Islands faces a triple burden of disease: communicable disease, non-communicable diseases, and the health impacts of climate change.^8^, ^105^ High-priority, climate-sensitive health risks in the Marshall Islands span insufficient food and water; vector-borne, non-communicable, and respiratory diseases; mental health stress; and extreme weather events.^106^ The health impacts of extreme weather events alone have the potential to be widespread and devastating, resulting in reduced drinking water and food availability, increased communicable diseases, and diminished mental health and wellbeing.^8^, ^107^ Despite relevant evidence showing the health risks associated with climate effects in the Marshall Islands, crucial gaps in research and evidence exist. Very little research has been conducted within the Marshall Islands, or the Pacific Islands more broadly.^105^, ^108^ As a result, there are no granular data on the health impacts of climate change experienced by minoritised people in the Marshall Islands, mirroring global epistemic injustice in this area. Research must identify climate-attributable changes to health indicators over time; no data of this nature currently exist for the Marshall Islands and are limited across the Pacific Islands.^105^ Research in these areas is crucial for guiding climate action and health policy.

Adaptation is also essential to addressing the health impacts of climate change. However, Marshallese adaptive capacity has been diminished by colonialism, creating an economy of dependency and limiting Marshallese sovereignty over adaptative planning due to power differentials.^109^ From the beginning of the 19th century, the Marshall Islands passed under colonial rule of Spain, Germany, Japan, and finally the USA. While under American occupation, the Marshall Islands additionally faced nuclear colonisation: the USA tested more than 65 nuclear weapons on the Bikini and Enewetak atolls, contaminating water and soil with long lasting implications for food access, forcibly displacing Indigenous communities and rendering 20% of the Marshall Islands uninhabitable.^107^, ^110^, ^111^ Racism was central to the justification of such violence.^6^ This same racist logic has facilitated atmospheric colonisation,^21^ causing the Marshall Islands to now face the existential threat posed by climate change. Prominent Marshallese climate activist and poet Kathy Jetnil-Kijiner has powerfully drawn this parallel between nuclear testing and climate change: “We're seen as disposable in both situations. We're disposable. Our lives don't matter, the war matters, nuclear bombs matter. Our lives don't matter, oil matters, money matters, gas matters, profits matter.”^112^

In the face of climate change and an apathetic global system, the Marshallese are not passive. They are spearheading global action on climate change through the Climate Vulnerable Forum and the High Ambition Coalition. As articulated by the Paris Agreement and Glasgow Pact, staying within 1·5°C of warming (which would still result in some damage) is paramount to the survival of the Marshall Islands and Large ocean states and require drastic emissions reduction. As the Marshallese are fighting for survival, the rich nations of the world, including Marshall Islands' former colonisers, must take responsibility for their actions by repairing past and ongoing harm.

### Health inequalities within countries

Climate change and other human-induced environmental changes, such as loss of biodiversity and air pollution, disproportionately affect the health of minoritised people globally. Indigenous Peoples live in different contexts, affected by different histories, and according to different lifeways. Despite these differences, Indigenous Peoples around the world bear an extraordinary burden of the health threats of climate change.^113^ This burden is due to a legacy of colonial subjugation, responsible for catastrophic loss of life, loss of lands, and ethnocide.^114^ In efforts to recognise the scope of land dispossession and forced removals in the USA, an investigation found that Indigenous communities have experienced near total loss of historical coextensive territorial homelands (98·9%), an average forced removal from territorial homelands of 239 km, and contemporary lands that have more heat days (ie, days when the maximum temperature surpasses approximately 38·8°C), less precipitation, and decreased mineral value potential than historical lands.^115^

Indigenous Peoples are also minoritised through evictions with no compensation for their relocation,^59^ subsequent financial burdens associated with housing insecurity and displacement,^59^, ^64^ and isolation and stress stemming from a disrupted spiritual and cultural relationship with their land.^62^, ^65^ Indigenous communities have been stewards of land and ecosystems, defending it against ongoing extraction from industry and managing an estimated 20–25% of the Earth's land and 80% of biodiversity, all while comprising only 5% of the world's population.^116^

## Discussion

### Interaction between climate change, health, and discrimination

This is the first scoping review that has summarised current evidence on how structural discrimination interacts with climate change to cause profound damage to minoritised people's health globally. The geographical visualisation has illustrated geopolitical inequities in responsibility for emissions: Global South countries (and minoritised people in any region) have contributed the least to the problem, yet experience the health impacts most acutely. Climate breakdown is being driven by processes of atmospheric colonisation and its consequences unfold along colonial lines. The case studies and policy analysis have elucidated the relationship between climate change, health, and discrimination, manifesting through extractive behaviours of powerful industries, often facilitated by governments. For example, government subsidies for fossil fuels exceed national health spending in some countries and totals US$400 billion globally.^3^ Applying racial justice to climate change and health reveals opportunities for the health community to redress unequal power dynamics between minoritised communities and the geopolitical and commercial factions that dominate the world economy and directly affect health. Racial justice necessitates repairing the harm through equitable distribution of the costs of adverse effects of climate change and resources to address climate change.

Our analysis of climate-related health inequities, and the parameters defining evidence-based solutions, are shaped by a research lens informed by data, policies, and practices produced by systems of colonialism and discrimination. For example, our understanding of climate change, health, and discrimination is limited by the concentration of studies on racial and ethnic health inequalities in North America, enacting epistemic injustice through both hypervisibility of some characteristics and erasure of those not accounted for. Geographical inequalities between countries are better documented due to greater data availability and policy attention.

Colonial legacies of profit and power are ever present today. As shown in the case studies and geographical visualisation, reliance on oil, gas, and other extractive industries that drive excessive emissions is a matter of life and death for minoritised people. Although millions of people in Europe face fuel poverty and residents in the Niger Delta are poisoned by oil spills, the companies responsible report soaring profits.^117^ Health institutions' investment in fossil companies continues despite overwhelming evidence that such companies are actively blocking climate justice and harming the health of minoritised people. A fair and just transition to renewable energy to meet the 1·5°C climate target requires transdisciplinary solidarity between movements fighting colonialism, racism, and health inequities.

### Relational Indigenous lifeways and health

Indigenous communities continue to contend with the ontological and epistemic assault of colonialism on Indigenous lifeways, cultures, languages, and spirituality, resulting in a profound cultural discontinuity that challenges the capacity to engage Indigenous Traditional Ecological Knowledges towards adaptation or mitigation efforts (or both).^118^, ^119^, ^120^ Meanwhile, the interdependence and kinship shared between local ecosystems, sacred places, and Indigenous Traditional Ecological Knowledges are persistently threatened by climate change, undermining community health.

Unequal health outcomes highlighted by existing evidence in the scoping review are perpetuated by political exclusion and under-representation of minoritised voices from decision-making spaces. Indigenous communities are continuously excluded from policy-making processes,^60^, ^64^ an enactment of colonial violence,^64^ and have few protections against intrusive developments by fossil fuel companies.^61^ Despite increasing recognition of Traditional Knowledges in climate adaptation and mitigation plans, these have been symbolic rather than applied practically on a global scale.^121^ Non-tokenistic representation of Indigenous voices in decision-making spaces is an important solution to alleviating these health inequalities, but is severely scarce.^60^, ^64^

In efforts to mitigate the intersecting and persistent threats of settler-colonialism, white supremacy, and structural discrimination, Indigenous communities have spearheaded movements to decolonise the Anthropocene across multiple sectors, namely public health policy, land rights, maternal and child health, and environmental health policy.^122^ Planetary health recognises the need to pursue mechanisms that confront the enduring impacts of structural discrimination while advancing the often-cited adaptive capacity of Indigenous Knowledge systems, and appropriately resourcing movements to decolonise and reclaim Indigenous cultures, lifeways, and traditional practices.^121^ Ultimately, allies and global health movements must recognise the crucial relationship and kinship of Indigenous communities with local ecosystems and associated ecological knowledge systems as a solution to climate change and representative of model sustainability practices.

### Reparative justice and human rights legal interventions

The evidence in this Health Policy shows the unequal distribution of responsibility for damage between countries, whereby the Global North is disproportionately responsible for damage and the Global South is least responsible. The Global North nations have colonised the atmospheric commons for their own enrichment through forms of industrialisation and growth that have relied on colonial patterns of appropriation.^88^ Across all countries, responsibility lies primarily with the affluent and ruling classes, due to their high levels of emissions^83^ and disproportionate control over energy systems and national legislation. In many settings, extraction lies more with corporations with substantial economic influence.^89^ Alternative economic paradigms such as degrowth, wellbeing economy, and doughnut economics—all rooted in ecological economics—offer opportunities to make the transition to an economic system that is designed to promote human and planetary health from the outset, rather than one where social and environmental damage must be constantly corrected afterwards.^123^

In light of the evidence discussed here, there is a clear health case for supporting climate reparations.^124^ Those countries, companies, and ruling classes who are most responsible both historically and currently should pay monetarily to the most affected peoples and areas and repair harm holistically.^125^ The health community must not only lobby to ensure that loss and damage funds are rapidly implemented, it must also demand more to heal and repair harm, grounded in principles of reparative justice.^126^ Reparative justice can take many forms: debt cancellation; giving land back to communities; unconditional grants; a Global Climate Stabilisation Fund according to responsibility; divestment from extractive industries; and transfer of technology, wealth, and resources.^127^ Scholars urge that reparations involve not only addressing past harm but a transformative worldbuilding project for ecological futures.^125^

As discriminatory policies are enabled by our current political and legal landscapes, a legal approach that employs human rights frameworks can provide grounds for the structural change that is necessary to address the deep health inequities outlined throughout this Health Policy. International human rights legal interventions can be a tool for challenging racist and discriminatory policies, through setting shared visions and commitments around the human right to health and enshrining mechanisms for public and private actors to be held accountable for violating equality frameworks.^128^ For example, health organisations can support the increasingly popular method of litigation against the fossil fuel industry to stop racist and discriminatory practices deployed by the industry to harm the health of minoritised groups. When international judicial systems fail communities, initiatives such as the People's Health Tribunal provides space for testimonies from the front lines of climate violence caused by extractive industry giants based in the Global North.^129^ This initiative is an example of the health community in the Global North practising solidarity-driven work with communities across the African continent and diaspora.

### Strengths and limitations

A key strength of this paper lies in its multiple methods, employing a scoping review, geographical visualisations, case studies from most affected peoples and communities, testimonies, and policy analysis. The geographical visualisation draws together a robust body of literature, visually complemented by original maps. These maps are the first to present unequal responsibility for climate change and health outcomes in this way. The inclusion of case studies and policy analysis sheds light on the complex pathways through which unequal responsibility leads to unequal impact, exacerbated by discrimination. Through these methods, we respond to calls for consideration of the multiple injustices and their health implications in climate and health.^130^

We encountered several methodological challenges highlighting the difficulty of analysing injustice with existing climate and health indicators.^25^ The literature represents scholarship on the unequal health impacts of climate change, a topic that is grossly underexamined due to the skewing of global research efforts towards the health needs of high-income countries. Additionally, focusing on papers written in English narrows the reach of our analysis and excludes non-English-speaking research communities. Further, the search strategy was limited to describing health inequities due to climate change but not the effects of drivers of climate change, such as consumerism and extractivism, or more broadly conceived health effects, such as wellbeing and social health. The scoping review did not capture the complexity of pathways linking climate change, discrimination, and health impacts, especially when either climate change or health were not explicitly discussed.

For the geographical visualisation, the availability of data for map creation were limited overall, as existing data initiatives do not capture information on variables that reflect different forms of discrimination and as a result are not presented in a granular and disaggregated manner to allow a more complete examination of climate, health, and discrimination. Different forms of inequality and discrimination could not be captured beyond the traditional geographical or economic and income inequalities. As such, the maps represent only regional-level inequalities. Further, existing data are probably an under-estimation as they only cover a select number of climate-related causes of death and do not include the health impacts of economic damage, major heat waves, river flooding, water scarcity, or conflict.

## Towards just futures

We conclude that a holistic approach to climate change and health requires not only addressing the web of social, commercial, and political determinants of health (eg, the built environment, education, and income) but going one step further into the oppressive structures that underpin all these conditions. Colleagues must strive to incorporate anti-racism, anti-xenophobia, and anti-discrimination in their work, centring voices of the most affected peoples and communities resisting the root causes of the health inequalities. As such, we have set out guiding principles to approach research and action at this intersection of climate change, health, and discrimination, at differing levels of society.

**Figure 3:**
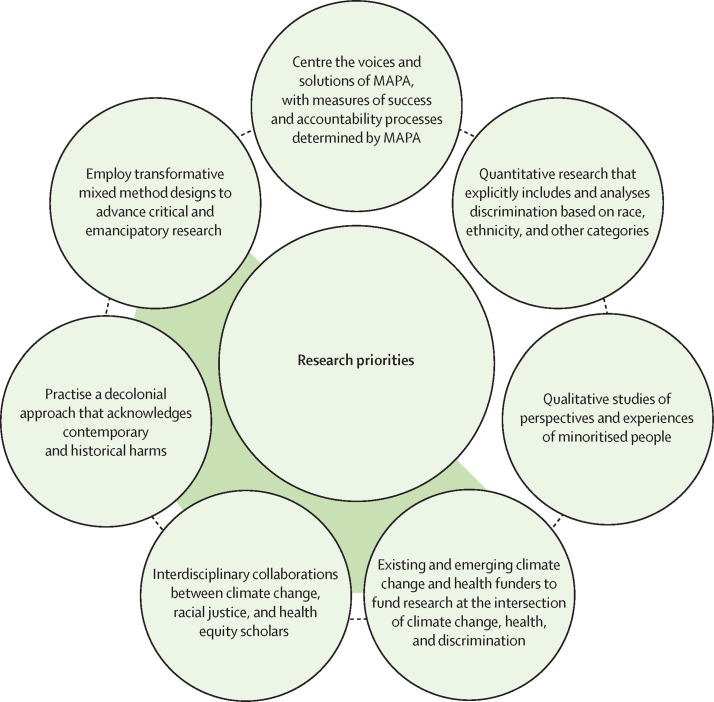
Research priorities at the nexus of climate change, health, and discrimination MAPA=most affected people and areas.

We invite scholars to pursue research that interrogates and repairs structural discrimination. The nexus of climate change, health, and discrimination offers a wealth of opportunities to engage with research that is justice-driven, has hyperlocal to international repercussions, and harnesses the potential in interdisciplinary collaborations to create policies and practices for equitable health outcomes and flourishing ecosystems. We present a non-exhaustive list of research priorities (figure 3). For example, we highlight the possibility for transformative mixed methods to address structural issues, including racism and inequity. Study designs that use participatory action research and restorative practices have been shown to be effective in flattening hierarchies when conducting research with minoritised populations, legitimising and contextualising perspectives, and promoting critical consciousness. Hegemonic research involving randomised controlled trials and epidemiological methods need valid critique over their appropriateness in this complex research context. Publications from a diversity of epistemologies and ontologies using alternative methods carry the opportunity for producing emancipatory research outcomes.^131^

We urge the health community, including everyone whose work affects health, to engage in the principles set out here (panel 4), based on Abubakar and colleagues'^128^ six principles for confronting the impact of discrimination on health from *The Lancet* Series on racism, xenophobia, discrimination, and health. Our values were developed from the findings of this paper and the expertise of the authors (see appendix p 20 for the process of developing the principles). We urge the health community to tackle root causes of historical and ongoing harm and trauma brought about when climate change interacts with structural discrimination.

Panel 4 **Principles to guide research and action on climate change, health, and racial justice**

The health community can offer skills, solidarity, research, and organising power and be a strong voice for justice. The health community's role in dismantling power structures to achieve health equity should be embraced at each level of society, aligned with Devakumar and colleagues'^6^ conceptual model from *The Lancet* Series on racism, xenophobia, discrimination, and health—ie, the individual, community, institutional, health system, and structural level (figure 1). In taking up these values, we urge the health community to be mindful of context and to be guided by communities taking action.

**Individual**

• Centre the voices and solutions of the most affected people and areas in policy, education, advocacy, and action to practise a decolonial approach
• Accountability for measures of success in policy and action should lie with the most affected people and areas

**Communities**

• Global North communities must practise allyship by creating space for Global South voices in decision making through non-tokenistic means
• Diverse forms of knowledge, including Indigenous Knowledges, should be embraced during decision making

**Institutions and health systems**

• Education institutions should raise critical consciousness about power imbalances and discrimination related to climate and health
• Unequal responsibility of powerful countries, institutions, systems, and industries must inform actions
• Health institutions must support the dismantling of extractive industries that sustain health inequities
• Develop and implement policies that take human rights-based approaches to health

**Structural**

• Support initiatives that foster healing and repair, including calls for reparations
• Take a solidarity-driven approach, bringing intersecting issues together such as gender and racial justice
• Take climate justice as the starting point for action, not an afterthought

## Search strategy and selection criteria

This literature review addressed the question: how does climate change lead to health inequalities by race, ethnicity, caste, Indigeneity, religion, migratory status, or skin colour? We ran search terms (appendix p 11) through six databases: MEDLINE, Embase, Web of Science, Scopus, Global Health, and GEOBASE, with no date restrictions on Dec 17, 2021. We restricted papers to availability in an English version. We also used subject headings related to the search terms in running the search, to ensure that no relevant literature was missed. The literature search returned 830 titles. The title and abstract of each paper were then independently screened against the inclusion criteria (appendix p 12) by two authors (TAD, CS, JB, MH, PdMS, RI, or SE) and any conflicts were resolved by a third author (SE or MH). Full-text screening was then done for 96 papers. Each paper was screened independently by two authors, and conflicts were resolved in discussion. 35 peer-reviewed articles met the inclusion criteria for this paper. All studies were published between 2003 and 2021, and both qualitative (n=15) and quantitative (n=20) studies were included. The studies were from North America (USA=22; Canada=1), Australasia (n=4), Africa (n=3), Asia (n=2), Pacific islands (n=2), and South America (n=1). A PRISMA diagram and checklist can be found in the appendix (p 13). We extracted and charted data with the following headings: author, title, journal, year, country, location, population affected, life course stage, study type, aims, methods, climate indicator, health indicator, inequity indicator, outcome, limitations, and summary. We specifically sought data on climate, health, and inequity indicators (variables) as identified by the included sources. Definitions of all inequity indicators can be found in the glossary of terms.^6^ All data extraction was verified by a second author.

## Declaration of interests

TAD, SS, PdMS, JB, SE, MH, RI, HM, HPN, CS, and DD report grants from the Wellcome Trust (224687/Z/21/Z) paid to University College London and for which DD is the principal investigator. The other authors declare no competing interests.
