# Supplementary material for: Envisioning environmental equity: climate change, health, and racial justice
Source: Lancet. 2023 Jul 1;402(10395):64–78. doi: 10.1016/S0140-6736(23)00919-4 (PMC10415673; doi:10.1016/S0140-6736(23)00919-4)
Supplement: Supplementary appendix [file mmc1.pdf]

THE LANCET

Supplementary appendix

This appendix formed part of the original submission and has been peer reviewed. We post it as supplied by the authors.

Supplement to: Deivanayagam TA, English S, Hickel J, et al. Envisioning environmental equity: climate change, health, and racial justice. *Lancet* 2023; published online May 29. https://doi.org/10.1016/S0140-6736(23)00919-4.

Appendices

Appendices **1**
Appendix 1 - Glossary of Terms 2
Appendix 2 - Scoping review results 6
Appendix 3 - Search terms 11
Appendix 4 - Inclusion and exclusion criteria 12
Appendix 5 - PRISMA diagram and checklist 12
Appendix 6 - Figure 1 with references 17
Appendix 7 - Representative Concentration Pathways (RCP) Descriptions 19
Appendix 8 - The process of developing principles to guide action 20

Appendix 1 - Glossary of Terms

Anthropocene

An unofficial unit of geologic time, used to describe the most recent period in Earth's history when human activity started to have a significant impact on the planet's climate and ecosystems.

Capitalism

An economic system characterised by highly inequitable distributional dynamics that benefit the elite few.[1] The capitalist economy is organised around and dependent on perpetually increasing aggregate production, the purpose of which is not primarily to meet human needs, but rather to maximise and accumulate profit.[1,2] Toward this end capitalism seeks to cheapen labour and resource inputs, which often entails processes of extraction and exploitation, including through historical and ongoing processes of colonisation, dispossession and coercion.

Colonialism

Colonialism is a practice of domination, which involves the subjugation of one people to another. The practice of colonialism usually involved the transfer of population to a new territory, where the arrivals lived as permanent settlers while maintaining political allegiance to their country of origin. Imperialism, which often accompanied colonialism, draws attention to the way that one country exercises power over another, whether through settlement, sovereignty, or indirect mechanisms of control. Colonialism was rationalised by a science of human difference built on a political belief that certain groups were inferior and naturally subservient.

Discrimination

Discrimination is differential treatment or outcomes that are unfavourable towards a group or an individual based on some aspect of their actual or perceived identity, such as race, religion, nationality, physical ability, gender, sexual orientation, class or social status.

Ethnicity

"Ethnicity is a social construct that categorises people based on characteristics like language spoken, values, cultural factors, behaviours, ancestral geography locations. There is overlap between racial and ethnic categories and structures of exclusion, given how groups of people who share ancestry, language and culture for example are also likely to share physical phenotypes."[3]

Global North and South

In our paper, we use the term global North to refer to the USA, Canada, Europe, Israel, Australia, New Zealand, and Japan. Global South refers to the rest of the world.[4] These terms emerged in response to the regional and transnational division of labour, resources, and capital, separating countries into core, semi-peripheral and peripheral economies.[5] The global South are the "peripheral" countries that tend to be previously colonised and remain socially and culturally minoritised.[6]

Intersectionality

A term coined by Kimberlé Crenshaw, refers to the interplay between social categorisations often thought of in silos, such as race, gender or class, overlapping and deepening interdependent systems of oppression and disadvantage (Shannon et al. 2022). It is the notion that discrimination can take many forms, and two

or more forms of discrimination can lead to greater than the sum of each type of discrimination in isolation.[7] Birthed from radical Black feminist, post-colonial and critical race theory, intersectionality aims to synergise various concerns of social justice, moving beyond individual factors .[8]

Minoritised

Minoritised is defined as 'individuals and populations, including numerical majorities, whose collective cultural, economic, political and social power has been eroded through the targeting of identity in active processes that sustain structures of hegemony'. When describing populations who experience racism, xenophobia and discrimination, it is important to recognise the active structural processes that have resulted in their discrimination.

Planetary boundary

In the scientific literature, this boundary has been defined as 350ppm concentration of CO_2 in the atmosphere.[9] This concept starts from the principle that the atmosphere is a commons, and that all people are entitled to use an equal share of it within the safe planetary boundary. Cumulative emissions exceeded this level in the late 1980s, driving the crisis of climate breakdown that is evident today. By dividing the carbon budget associated with this boundary equitably among nations according to their share of the global population, averaged over time, it is possible to determine the extent to which nations have exceeded their fair share of the safe budget, and thus how much they have contributed to pushing global emissions beyond the planetary boundary. National responsibility for climate change is calculated using this 'fair share' approach.

Race

"Race is a socially constructed classification that relies on someone's actual or perceived physical appearance and ancestry, and the social, economic and political meaning that is imbued in these. Race was constructed and functions as an essential tool of racism, to separate and create racial hierarchy. The modern history of race, centrally features its use as a mechanism for assigning superiority and inferiority, and determining access to resources and human rights, despite racial hierarchies being morally and biologically baseless. Racial categories are fluid non-discrete variables, and the meaning of race can change over time, location and context. These social racial categories are embedded in and function as tools of broader intersecting networks of oppression (such as patriarchy) - operating through social, legal, economic, political and health structures to differentially allocate privilege and power."[3]

Racial capitalism

Coined by Robinson, this is an exploitative process, where economic and social value is extracted based on the racial identity of a person. Where there was formation of a capitalist society with winners and losers, it "pursued essentially racial directions, so too did social ideology". Ideologies of race and other forms of difference have long been leveraged to justify capitalist processes.

Racism

Racism is an organised system affording power and privilege in accordance with an established racial hierarchy, where the social construction of racial categories and thus racialisation is used to maintain this hierarchy. Racism operates to protect the rights, power and livelihoods of those at the top of this created hierarchy whilst placing those towards the lower rungs in closer proximity to death. Structural racism is at

the core of other forms of racism, centring separation and hierarchical power, describing the socioecological macro level processes and systems in which people and institutions exist, which maintain and perpetuate racial inequity, including through facially race-neutral means.

Redlining
A policy of systematic disinvestment in minority and low-income neighbourhoods by financial and other services, utilised in countries such as the USA.

Xenophobia
Xenophobia is the fear or hatred of, or discrimination against those who are considered to be Foreigners. The targets of xenophobia are often groups or individuals perceived as outsiders to the nation and undeserving of the benefits associated with citizenship or national membership.

1 Moore JW. Capitalism in the Web of Life: Ecology and the Accumulation of Capital. Verso Books, 2015.

2 Robinson CJ. Black Marxism: The Making of the Black Radical Tradition. Labour / Le Travail. 1985; **16**: 363.

3 Devakumar D, Selvarajah S, Abubakar I, *et al.* Racism, xenophobia, discrimination, and the determination of health. *Lancet* 2022; **400**: 2097–108.

4 Hickel J. Quantifying national responsibility for climate breakdown: an equality-based attribution approach for carbon dioxide emissions in excess of the planetary boundary. *Lancet Planet Health* 2020; **4**: e399–404.

5 Independent Commission on International Development Issues, Brandt W. North-South, a Programme for Survival: Report of the Independent Commission on International Development Issues. London : Pan Books, 1980.

6 Wallerstein IM, Wallerstein SRI. World-systems Analysis: An Introduction. Duke University Press, 2004.

7 Crenshaw K. Mapping the Margins: Intersectionality, Identity Politics, and Violence against Women of Color. Stanford Law Review. 1991; **43**: 1241.

8 Kapilashrami A, Hankivsky O. Intersectionality and why it matters to global health. *Lancet* 2018; **391**: 2589–91.

9 Steffen W, Richardson K, Rockström J, *et al.* Sustainability. Planetary boundaries: guiding human development on a changing planet. *Science* 2015; **347**: 1259855.

Appendix 2 - Scoping review results

Categorisation	Health outcome	Impacts of climate change on health
**Indigeneity**	Nutrition	● Uganda and Peru: Due to increased weather variability, extreme events and gradual temperature changes, climate change has negatively impacted food security and food availability for Batwa indigenous populations in Uganda and the Shawi in the Peruvian Amazon [1–3]
	Maternal and child health	• USA: Indigenous women reported a rise in fertility concerns, miscarriages, eclampsia, pre-term birth, and gynaecological cancers which they associated with deteriorating health of the environment around them[4]<br>● Uganda: Through negatively impacting agricultural productivity and financial security, climate change negatively impacted maternal and child health of Batwa mothers and children [5]
	Communicable disease	● Uganda: Increases in malaria outbreaks have become a concern amongst Batwa communities due to higher levels of precipitation and more resulting stagnant water [2]
	Mental Health	● Tuvalu: Higher stress levels in response to the immediate impacts of climate change such as extreme weather, and the longer-term impacts such as food shortages, potential relocation, and financial hardships, and potential impacts on future generations [6]<br>● USA: Displacement due to the climate crisis leads to stress and isolation, resulting in a disrupted relationship between land/environment and community. This disruption has caused cultural losses, with emphasis on the preservation of and connection to ancestral knowledge and/or roots [7]
	Respiratory	● Australia: Indigenous Australians in the Northern Territory experience higher rates of hospital admissions for respiratory disease than White Australians [8]
**Migratory Status**	Heat-related health impacts	● USA and Austria: Migrants are disproportionately exposed to heat leading to heat-related health issues [9,10]<br>● Australia: migrants are more vulnerable to heat-related health problems, in part due to a fear of "standing out" in public cooled spaces like malls [11]
	Occupational health	● USA: Undocumented Latino/a and Indigenous migrants in the US were more vulnerable to health impacts of wildfires due to lack of accessibility of disaster risk information, and hazardous work conditions [12]
	Nutrition	● For climate-induced Tuvaluan migrants New Zealand and Tuvalu, migration/relocation led to increased consumption of processed foods, and increased weight gain, diabetes, and tooth decay. Financial constraints also restricted access to healthier foods for Tuvaluan migrants in New Zealand [13–15]
	Disease risk	● Pakistan: Migrants experiencing internal displacement due to climate-change related flooding reported an increase in gastrointestinal and respiratory diseases due to proximity to livestock during displacement [16]<br>● Bangladesh: internal migrants of lower socioeconomic standing were more likely to settle in flood-prone areas, increasing their exposure to flood risk [17]<br>● USA: Where proximity to water-based amenities is not indicative of high socioeconomic standing, Hispanic immigrants are exposed to greater flood risk [18]
	Restricted access to healthcare	● Linguistic and financial barriers and differences between receiving country and home country healthcare systems restrict migrant's access to healthcare in Australia, New Zealand, and Austria, leading to poorer climate-sensitive health outcomes for migrants [9,11,13,14]
**Race**	Heat-related illness and mortality	● USA: Top deciles of Latinx neighbourhoods by population in southwestern USA average 2.2C hotter than neighbourhoods in the lowest decile of Latinx populations [19]<br>● USA: In urban areas of the US and Puerto Rico, non-Hispanic Blacks were more likely to live in heat-risk related land cover areas, followed by Asians and Hispanics [20]<br>● USA: In Portland, Oregon, census blocks with higher proportions of non-white residents experience greater heat exposure than blocks with a greater proportion of white residents [21]<br>● USA: In Baltimore, Dallas and Kansas City, temperatures were higher on average in areas previously targeted for redlining, and these areas had higher proportions of Hispanic, African American, or both, residents [10].

		• USA: Land surface temperatures and heat-related ER visits in historically more-redlined areas were higher in Texas than less redlined areas [22]<br>• USA: A stronger temperature dependence in heat related deaths amongst Blacks than whites was found in seven US cities [23]<br>• USA: Black and Latino workers are underrepresented in HRI worker compensation data, but overrepresented in heat related illness worker compensation claims [24]<br>• Hispanics were less likely to have access to AC than Whites, but that this did not significantly impact hospitalisations [25]<br>• USA: Black households in NYC were twice as likely not to own AC when compared to white households, even after adjusting for income and risk perception of heat [26]<br>• Canada: No correlations between proportions of visible minorities and heat exposure at the census block level, possibly due to gentrification and lower levels of segregation in Montreal [27]
	Cardiorespiratory health	• USA: Association between daily max temperatures and paediatric emergency department visits for asthma was significantly stronger for African American than White children in Atlanta, Georgia [28]<br>• USA: Black people had the highest age-adjusted admission rates for ischaemic heart disease and asthma, both of which are climate sensitive. Asthma incidence and the magnitude of inequality will increase into 2040-2050, with Blacks still suffering the highest burden of disease. [29]<br>• USA: Significant association between non-optimum high and low temperatures and cardio-respiratory mortality and higher burden of temperature related cardiorespiratory in areas with a lower proportion of white inhabitants. [30]<br>• USA: African Americans were more likely to be hospitalised for asthma and acute myocardial infarction, but no association between PM2.5, PM10, or ozone and asthma- or AMI- related hospitalisations in Washington DC. [31]
	Maternal and newborn health	• USA: Increased heat exposure leads to significantly more prenatal hospitalisations in Black mothers compared to White mothers [32]<br>• USA: A higher percentage of PTBs occurred in minority races and Hispanics following summer heatwaves, and those children born to Black women with college degrees experienced a higher relative risk of PTB compared to White women with college degrees [33]
	Mental illness	• USA: Being Black or Hispanic was associated with a 403% increase in the odds of having a PTS score greater than 40 on the Post-Traumatic Stress Disorder Checklist, indicating PTS, following Hurricane Harvey [34]
	Vector-borne diseases	• USA: In ethnographic research in Detroit, Michigan, residents expressed concern about vector-borne diseases from rats and mosquitoes and that the concerns of non-white and low-income residents were not taken into consideration by green infrastructure projects [35]

References

1 Zavaleta C, Berrang-Ford L, Ford J, *et al.* Multiple non-climatic drivers of food insecurity reinforce climate change maladaptation trajectories among Peruvian Indigenous Shawi in the Amazon. *PLoS One* 2018; **13**: e0205714.

2 Berrang-Ford L, Dingle K, Ford JD, *et al.* Vulnerability of indigenous health to climate change: a case study of Uganda's Batwa Pygmies. *Soc Sci Med* 2012; **75**: 1067–77.

3 Bryson JM, Patterson K, Berrang-Ford L, *et al.* Seasonality, climate change, and food security during pregnancy among indigenous and non-indigenous women in rural Uganda: Implications for maternal-infant health. *PLoS One* 2021; **16**: e0247198.

4 Liddell JL, Kington SG. 'Something Was Attacking Them and Their Reproductive Organs': Environmental Reproductive Justice in an Indigenous Tribe in the United States Gulf Coast. International Journal of Environmental Research and Public Health. 2021; **18**: 666.

5 MacVicar S, Berrang-Ford L, Harper S, *et al.* How seasonality and weather affect perinatal health: Comparing the experiences of indigenous and non-indigenous mothers in Kanungu District, Uganda. *Soc Sci Med* 2017; **187**: 39–48.

6 Gibson K, Haslam N, Kaplan I. Distressing encounters in the context of climate change: Idioms of distress, determinants, and responses to distress in Tuvalu. *Transcult Psychiatry* 2019; **56**: 667–96.

7 Johnson-Jennings M, Billiot S, Walters K. Returning to Our Roots: Tribal Health and Wellness through Land-Based Healing. Genealogy. 2020; 4: 91.

8 Green D, Bambrick H, Tait P, *et al.* Differential Effects of Temperature Extremes on Hospital Admission Rates for Respiratory Disease between Indigenous and Non-Indigenous Australians in the Northern Territory. *Int J Environ Res Public Health* 2015; **12**: 15352–65.

9 Wiesböck L, Wanka A, Mayrhuber EA-S, *et al.* Heat Vulnerability, Poverty and Health Inequalities in Urban Migrant Communities: A Pilot Study from Vienna. Climate Change Management. 2016; : 389–401.

10 Wilson B. Urban Heat Management and the Legacy of Redlining. Journal of the American Planning Association. 2020; **86**: 443–57.

11 Hansen A, Nitschke M, Saniotis A, *et al.* Extreme heat and cultural and linguistic minorities in Australia: perceptions of stakeholders. *BMC Public Health* 2014; **14**: 550.

12 Méndez M, Flores-Haro G, Zucker L. The (in)visible victims of disaster: Understanding the vulnerability of undocumented Latino/a and indigenous immigrants. Geoforum. 2020; **116**: 50–62.

13 Paul EJ, O'Brien S, Nosa V, Terry TE, Goldman R. Characterizing the health experience of Tuvaluan migrants in Auckland, New Zealand. *International Journal of Migration, Health and Social Care* 2021; **17**: 508–24.

14 Emont J, Anandarajah G. Rising Waters and a Smaller Island: What Should Physicians Do for Tuvaluans? *AMA Journal of Ethics* 2017; **19**: 1211–21.

15 McMichael C, Powell T. Planned Relocation and Health: A Case Study from Fiji. International Journal of Environmental Research and Public Health. 2021; **18**: 4355.

16 Braam DH, Chandio R, Jephcott FL, Tasker A, Wood JLN. Disaster displacement and zoonotic disease dynamics: The impact of structural and chronic drivers in Sindh, Pakistan. PLOS Global Public Health. 2021; 1: e0000068.

17 Liu Z, Balk D. Urbanisation and differential vulnerability to coastal flooding among migrants and

nonmigrants in Bangladesh. *Popul Space Place* 2020; **26**. DOI:10.1002/psp.2334.

18 Maldonado A, Collins TW, Grineski SE, Chakraborty J. Exposure to Flood Hazards in Miami and Houston: Are Hispanic Immigrants at Greater Risk than Other Social Groups? *Int J Environ Res Public Health* 2016; **13**. DOI:10.3390/ijerph13080775.

19 Dialesandro J, Brazil N, Wheeler S, Abunnasr Y. Dimensions of thermal inequity: Neighborhood social demographics and urban heat in the Southwestern U.s. *Int J Environ Res Public Health* 2021; **18**: 941.

20 Jesdale BM, Morello-Frosch R, Cushing L. The Racial/Ethnic Distribution of Heat Risk–Related Land Cover in Relation to Residential Segregation. Environmental Health Perspectives. 2013; **121**: 811–7.

21 Voelkel J, Hellman D, Sakuma R, Shandas V. Assessing Vulnerability to Urban Heat: A Study of Disproportionate Heat Exposure and Access to Refuge by Socio-Demographic Status in Portland, Oregon. *Int J Environ Res Public Health* 2018; **15**. DOI:10.3390/ijerph15040640.

22 Li D, Newman GD, Wilson B, Zhang Y, Brown RD. Modeling the Relationships Between Historical Redlining, Urban Heat, and Heat-Related Emergency Department Visits: An Examination of 11 Texas Cities. *Environ Plan B Urban Anal City Sci* 2022; **49**: 933–52.

23 O'Neill MS, Zanobetti A, Schwartz J. Modifiers of the Temperature and Mortality Association in Seven US Cities. *Am J Epidemiol* 2003; **157**: 1074–82.

24 Hesketh M, Wuellner S, Robinson A, Adams D, Smith C, Bonauto D. Heat related illness among workers in Washington State: A descriptive study using workers' compensation claims, 2006-2017. *Am J Ind Med* 2020; **63**: 300–11.

25 Guirguis K, Basu R, Al‐Delaimy WK, *et al.* Heat, Disparities, and Health Outcomes in San Diego County's Diverse Climate Zones. GeoHealth. 2018; 2: 212–23.

26 Madrigano J, Lane K, Petrovic N, Ahmed M, Blum M, Matte T. Awareness, Risk Perception, and Protective Behaviors for Extreme Heat and Climate Change in New York City. *Int J Environ Res Public Health* 2018; **15**. DOI:10.3390/ijerph15071433.

27 Fan JY, Sengupta R. Montreal's environmental justice problem with respect to the urban heat island phenomenon. The Canadian Geographer / Le Géographe canadien. 2021. DOI:10.1111/cag.12690.

28 O'Lenick CR, Winquist A, Chang HH, *et al.* Evaluation of individual and area-level factors as modifiers of the association between warm-season temperature and pediatric asthma morbidity in Atlanta, GA. *Environ Res* 2017; **156**: 132–44.

29 McDonald YJ, Grineski SE, Collins TW, Kim Y-A. A scalable climate health justice assessment model. *Soc Sci Med* 2015; **133**: 242–52.

30 Zhang Y, Xiang Q, Yu Y, Zhan Z, Hu K, Ding Z. Socio-geographic disparity in cardiorespiratory mortality burden attributable to ambient temperature in the United States. *Environ Sci Pollut Res Int* 2019; **26**: 694–705.

31 Anderko L, Davies-Cole J, Strunk A. Identifying populations at risk: interdisciplinary environmental climate change tracking. *Public Health Nurs* 2014; **31**: 484–91.

32 Kim J, Lee A, Rossin-Slater M. What to Expect When It Gets Hotter: The Impacts of Prenatal Exposure to Extreme Heat on Maternal Health. 2019. DOI:10.3386/w26384.

33 Smith ML, Luke Smith M, Hardeman RR. Association of Summer Heat Waves and the Probability of Preterm Birth in Minnesota: An Exploration of the Intersection of Race and Education. International Journal of Environmental Research and Public Health. 2020; **17**: 6391.

34 Flores AB, Collins TW, Grineski SE, Chakraborty J. Disparities in Health Effects and Access to Health Care Among Houston Area Residents After Hurricane Harvey. *Public Health Rep* 2020; **135**: 511–23.

35 Carmichael C, Danks C, Vatovec C. Green Infrastructure Solutions to Health Impacts of Climate Change: Perspectives of Affected Residents in Detroit, Michigan, USA. Sustainability. 2019; **11**: 5688.

Appendix 3 - Search terms

1) Climate Change	2) Health	4) Inequalities	4) Form of discrimination
“Climate change” OR “Climate crisis” OR “Climate justice” OR “climate breakdown” OR “global warming” OR “global heating”	“Health” OR “disease” OR “illness” OR “morbidit*” OR “mortalit*” OR “life expectancy” OR “death”	“inequalit*” OR “disparit* OR “inequit*” OR “unequal” OR “discrim*” OR ”prejudic*”	“race” OR “racism” OR “ethnic*” OR “skin color” OR “skin colour” OR “skin tone” OR “BME” OR “BAME” OR “ethnic minorit*” OR “environmental racism” OR<br>“Migrant*” OR “refugee*” OR “asylum seek*” OR “immigrant*” OR “immigrat*” OR “discrim*” OR ‘prejudic*’ OR “xenophobi*” OR “antisemit*” OR “anti semit*” OR “islamophobi*” OR “caste” OR “indigen*” or “aboriginal*” OR “first nation*” OR “first peopl*”

Appendix 4 - Inclusion and exclusion criteria

Inclusion	Exclusion
I. Studies that report on the relation between:<br>A. Climate change, global heating, climate crisis<br>B. Health inequalities<br>C. Form of discrimination: Race/ ethnicity/ caste/ migratory status /indigeneity<br>II. Quantitative studies, including for example cross-sectional studies, cohort studies, case-control studies, interrupted time series. Studies must quantify unequal health impacts in minoritised group vs less-minoritised group e.g. mortality rate for White vs non-White population<br>III. Qualitative studies, including for example participatory research, case studies, ethnographies - can be included even if no comparator group<br>IV. Mixed-method studies	I. Non-human studies<br>II. Grey literature, editorials, conference proceedings, and studies that lack access to the full text<br>III. Reviews

Appendix 5 - PRISMA diagram and checklist

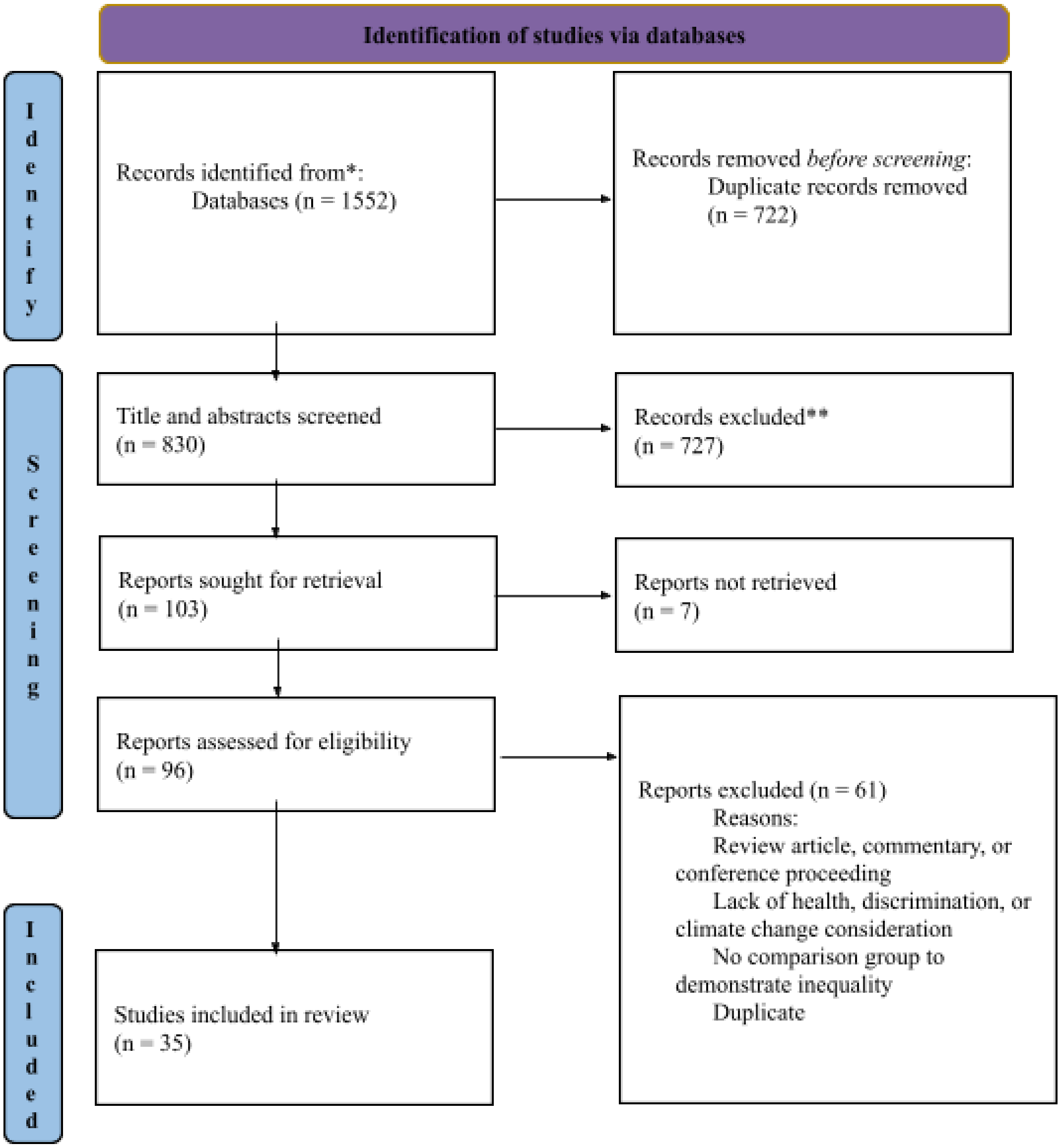

From: Page MJ, McKenzie JE, Bossuyt PM, Boutron I, Hoffmann TC, Mulrow CD, et al. The PRISMA 2020 statement: an updated guideline for reporting systematic reviews. BMJ 2021;372:n71. doi: 10.1136/bmj.n71

For more information, visit: http://www.prisma-statement.org/

Preferred Reporting Items for Systematic reviews and Meta-Analyses extension for Scoping Reviews (PRISMA-ScR) Checklist

SECTION	ITEM	PRISMA-ScR CHECKLIST ITEM	REPORTED ON PAGE #
**TITLE**			
Title	1	Identify the report as a scoping review.	2
**ABSTRACT**			
Structured summary	2	Provide a structured summary that includes (as applicable): background, objectives, eligibility criteria, sources of evidence, charting methods, results, and conclusions that relate to the review questions and objectives.	1
**INTRODUCTION**			
Rationale	3	Describe the rationale for the review in the context of what is already known. Explain why the review questions/objectives lend themselves to a scoping review approach.	2
Objectives	4	Provide an explicit statement of the questions and objectives being addressed with reference to their key elements (e.g., population or participants, concepts, and context) or other relevant key elements used to conceptualise the review questions and/or objectives.	4
**METHODS**			
Protocol and registration	5	Indicate whether a review protocol exists; state if and where it can be accessed (e.g., a Web address); and if available, provide registration information, including the registration number.	N/A
Eligibility criteria	6	Specify characteristics of the sources of evidence used as eligibility criteria (e.g., years considered, language, and publication status), and provide a rationale.	Appendix
Information sources*	7	Describe all information sources in the search (e.g., databases with dates of coverage and contact with authors to identify additional sources), as well as the date the most recent search was executed.	4
Search	8	Present the full electronic search strategy for at least 1 database, including any limits used, such that it could be repeated.	Appendix

Selection of sources of evidence†	9	State the process for selecting sources of evidence (i.e., screening and eligibility) included in the scoping review.	4; Appendix
Data charting process‡	10	Describe the methods of charting data from the included sources of evidence (e.g., calibrated forms or forms that have been tested by the team before their use, and whether data charting was done independently or in duplicate) and any processes for obtaining and confirming data from investigators.	4
Data items	11	List and define all variables for which data were sought and any assumptions and simplifications made.	See Devakumar et. al., 2022
Critical appraisal of individual sources of evidence§	12	If done, provide a rationale for conducting a critical appraisal of included sources of evidence; describe the methods used and how this information was used in any data synthesis (if appropriate).	N/A
Synthesis of results	13	Describe the methods of handling and summarising the data that were charted.	2
**RESULTS**			
Selection of sources of evidence	14	Give numbers of sources of evidence screened, assessed for eligibility, and included in the review, with reasons for exclusions at each stage, ideally using a flow diagram.	Appendix
Characteristics of sources of evidence	15	For each source of evidence, present characteristics for which data were charted and provide the citations.	Appendix
Critical appraisal within sources of evidence	16	If done, present data on critical appraisal of included sources of evidence (see item 12).	N/A
Results of individual sources of evidence	17	For each included source of evidence, present the relevant data that were charted that relate to the review questions and objectives.	4-7; Appendix
Synthesis of results	18	Summarise and/or present the charting results as they relate to the review questions and objectives.	4-7
**DISCUSSION**			

Summary of evidence	19	Summarise the main results (including an overview of concepts, themes, and types of evidence available), link to the review questions and objectives, and consider the relevance to key groups.	7
Limitations	20	Discuss the limitations of the scoping review process.	6-7, 12
Conclusions	21	Provide a general interpretation of the results with respect to the review questions and objectives, as well as potential implications and/or next steps.	7, 11-13
**FUNDING**			
Funding	22	Describe sources of funding for the included sources of evidence, as well as sources of funding for the scoping review. Describe the role of the funders of the scoping review.	13

JBI = Joanna Briggs Institute; PRISMA-ScR = Preferred Reporting Items for Systematic reviews and Meta-Analyses extension for Scoping Reviews.

From: Tricco AC, Lillie E, Zarin W, O'Brien KK, Colquhoun H, Levac D, et al. PRISMA Extension for Scoping Reviews (PRISMAScR): Checklist and Explanation. Ann Intern Med. 2018;169:467–473. doi: 10.7326/M18-0850.

Appendix 6 - Figure 1 with references

Individual health outcomes
- Climate change increases food insecurity and undernutrition, including for Batwa Indigenous communities in Uganda.[1]
- Following hurricane Harvey, Black Texas residents had a fourfold higher risk of PTSD that White residents.[2]
- Racially minoritised individuals face a disproportionate burden of occupational health risks such as heat-related illness.[3]

Communities and spatial determination
- Previously colonised countries are disproportionately located in the areas most impacted by climate change.
- Within USA cities, areas with greater proportions of Black, Hispanic, and Asian residents are hotter on average than areas with more White residents.[4,5]
- Rural-urban migrants in Dhaka, Bangladesh are more likely to land in areas of the city that are prone to flooding.[6]
- In Pakistan, exposure to flooding increased the proximity between those displaced to zoonotic and vector-borne diseases.[7]

Health systems
- Previously colonised countries have less economic resources to adapt their health systems in response to climate change, contributing to a disproportionate burden of deaths in the global South.[8]
- Without legal recognition or protection, climate refugees are excluded from accessing health systems in receiving countries.[9,10]
- Climate change is a cause of ill health; as demonstrated by the tragic death of Ella Addo-Kissi Debrah.
- Health systems must tackle the interwoven roots of discrimination that exist alongside their carbon emitting processes.

Institutions and systems
- The harmful health impacts of the fossil fuel industry affect minoritised populations even prior to reaching the high emissions stage by polluting land and water, eg. the oil spills in the Niger Delta.[11]
- Health institutions must support the dismantling of extractive industries that create health inequalities.

Intersecting systems of oppression
- Racially minoritised people with disabilities face unique challenges to disaster- and extreme weather-related evacuation, exacerbating health inequalities.[2]
- Climate change compounds racialised and gendered health harms, including increasing prenatal hospitalisation and preterm birth in racially minoritised women.[12,13]
- Racially minoritised people are overrepresented in low income groups who have limited financial capacity to mitigate climate-related health risks, including access to air conditioning.[14,15]

Structural discrimination
- Global capitalism has an extractive relationship with the global South, shaping local inequalities especially for Indigenous and rural communities.
- Settler colonialism institutionalises manufactured difference, creating political, social, and economic implications for those in the margins, e.g. life expectancy gap between Aboriginal and White Australians that is exacerbated by climate change.[16]
- Within a capitalist system, individuals and institutions are incentivised to project an image of tackling climate change or racial equity, being tokenistic and performative.

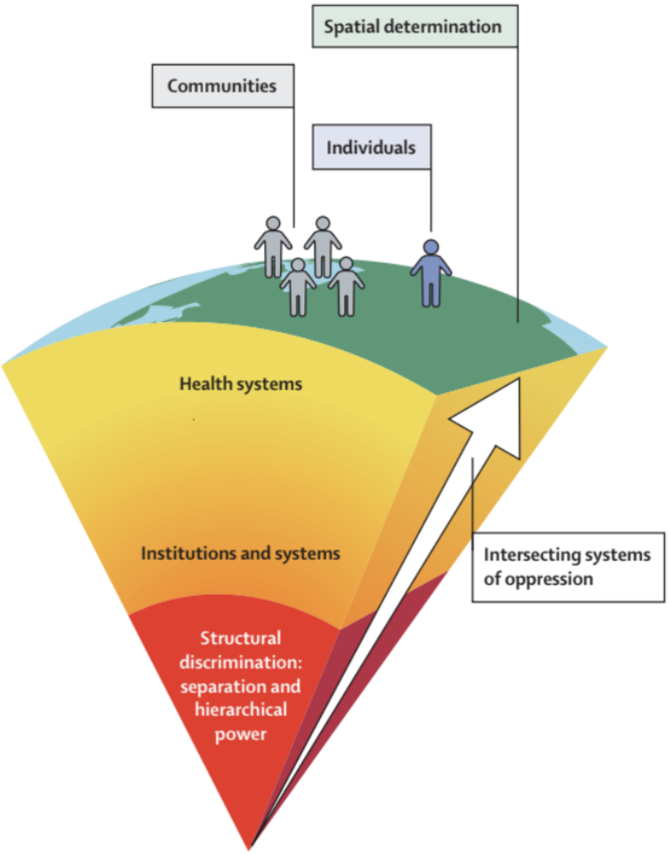

References

1. Ford JD, Edge V, Bryson JM, *et al.* Seasonality, climate change, and food security during pregnancy among indigenous and non-indigenous women in rural Uganda: Implications for maternal-infant health. 2021. https://repositorio.upch.edu.pe/handle/20.500.12866/9118.
2. Flores AB, Collins TW, Grineski SE, Chakraborty J. Disparities in Health Effects and Access to Health Care Among Houston Area Residents After Hurricane Harvey. *Public Health Rep* 2020; **135**: 511–23.
3. Hesketh M, Wuellner S, Robinson A, Adams D, Smith C, Bonauto D. Heat related illness among workers in Washington State: A descriptive study using workers' compensation claims, 2006-2017. *Am J Ind Med* 2020; **63**: 300–11.
4. Dialesandro J, Brazil N, Wheeler S, Abunnasr Y. Dimensions of thermal inequity: Neighborhood social demographics and urban heat in the Southwestern U.s. *Int J Environ Res Public Health* 2021; **18**: 941.
5. Voelkel J, Hellman D, Sakuma R, Shandas V. Assessing Vulnerability to Urban Heat: A Study of Disproportionate Heat Exposure and Access to Refuge by Socio-Demographic Status in Portland, Oregon. *Int J Environ Res Public Health* 2018; **15**. DOI:10.3390/ijerph15040640.
6. Liu Z, Balk D. Urbanisation and differential vulnerability to coastal flooding among migrants and nonmigrants in Bangladesh. *Popul Space Place* 2020; **26**. DOI:10.1002/psp.2334.
7. Braam DH, Chandio R, Jephcott FL, Tasker A, Wood JLN. Disaster displacement and zoonotic disease dynamics: The impact of structural and chronic drivers in Sindh, Pakistan. PLOS Global Public Health. 2021; **1**: e0000068.
8. Carleton TA, Jina A, Delgado MT, *et al.* Valuing the Global Mortality Consequences of Climate Change Accounting for Adaptation Costs and Benefits. 2020; published online July. DOI:10.3386/w27599.
9. Paul EJ, O'Brien S, Nosa V, Terry TE, Goldman R. Characterizing the health experience of Tuvaluan migrants in Auckland, New Zealand. *International Journal of Migration, Health and Social Care* 2021; **17**: 508–24.
10. Emont J, Anandarajah G. Rising Waters and a Smaller Island: What Should Physicians Do for Tuvaluans? *AMA Journal of Ethics* 2017; **19**: 1211–21.
11. Adekola J, Fischbacher-Smith M, Fischbacher-Smith D, Adekola O. Health risks from environmental degradation in the Niger Delta, Nigeria. Environment and Planning C: Politics and Space. 2017; **35**: 334–54.
12. Smith ML, Hardeman RR. Association of Summer Heat Waves and the Probability of Preterm Birth in Minnesota: An Exploration of the Intersection of Race and Education. *Int J Environ Res Public Health* 2020; **17**. DOI:10.3390/ijerph17176391.

13. Kim J (june), Lee A, Rossin-Slater M. What to Expect When It Gets Hotter: The Impacts of Prenatal Exposure to Extreme Heat on Maternal and Infant Health. 2019.
14. Madrigano J, Lane K, Petrovic N, Ahmed M, Blum M, Matte T. Awareness, Risk Perception, and Protective Behaviors for Extreme Heat and Climate Change in New York City. *Int J Environ Res Public Health* 2018; **15**. DOI:10.3390/ijerph15071433.
15. Guirguis K, Basu R, Al‐Delaimy WK, *et al.* Heat, Disparities, and Health Outcomes in San Diego County's Diverse Climate Zones. GeoHealth. 2018; **2**: 212–23.
16. Green D, Bambrick H, Tait P, *et al.* Differential Effects of Temperature Extremes on Hospital Admission Rates for Respiratory Disease between Indigenous and Non-Indigenous Australians in the Northern Territory. *Int J Environ Res Public Health* 2015; **12**: 15352–65.

Appendix 7 - Representative Concentration Pathways (RCP) Descriptions

In this paper, climate change scenarios are referred to as low emissions scenario, intermediate-1 emissions scenario, intermediate-2 emissions scenario, and high emissions scenario when they correspond to a Representative Concentration Pathway (RCP). These descriptive terms are employed for ease of interpretation of climate change scenarios by audiences that may not be familiar with the RCPs.

RCPs are time series emission and concentration scenarios that refer to the concentration of greenhouse gases, aerosols and chemically active gases, and land use and cover1. All pathways extend up to 2100 and are employed by the International Panel on Climate Change to project the impacts of climate change in the 21st century across a plausible range of human-caused forcings, or drivers of climate change2. Each RCP is defined according to the approximate radiative forcing in the year 2100 relative to 1750 3. See below for a brief description of each RCP:

Description used in this paper	RCP	Approximate Radiative Force	Impacts and Policy Description
Low Emissions Scenario	RCP 2.6	2.6 W m-2	Projected warming relative to 1850: unlikely to exceed 2C[3]<br>Scenario consistent with very low greenhouse gas emissions and the most stringent climate policy[4]
Medium Emissions Scenario A	RCP 4.5	4.5 W m-2	Projected warming relative to 1850: more likely than not to exceed 2C[3]<br>This scenario can be considered a very-low baseline[4]
Medium Emissions Scenario B	RCP 6.0	6.0 W m-2	Projected warming relative to 1850: likely to exceed[3]<br>This scenario can be considered a medium baseline scenario[4]<br>This RCP is not referred to in the paper.
High Emissions Scenario	RCP 8.5	8.5 W m-2	Projected warming relative to 1850: likely to exceed 2C, about as likely as not to exceed 4C[3]<br>High energy use scenario consistent with the absence of stringent climate mitigation[4]

1. IPCC DDC Glossary. https://www.ipcc-data.org/guidelines/pages/glossary/glossary_r.html (accessed May 26, 2022).
2. Chapter 1 — Global Warming of 1.5 oC. https://www.ipcc.ch/sr15/chapter/chapter-1/ (accessed May 26, 2022).
3. Intergovernmental Panel on Climate Change. Climate Change 2013 – The Physical Science Basis: Working Group I Contribution to the Fifth Assessment Report of the Intergovernmental Panel on Climate Change. Cambridge University Press, 2014.
4. van Vuuren DP, Edmonds J, Kainuma M, *et al.* The representative concentration pathways: an overview. *Clim Change* 2011.

Appendix 8 - The process of developing principles to guide action

The Principles to Guide Action are drawn from the literature and analysis presented in this paper, and the expertise of the authorship team. We decided to frame these recommendations as Principles to Guide Action in acknowledgement of the breadth of each point; each point can have many applications in practice. Further, Principles to Guide Action better describes the value-based nature of them, and acknowledges that these principles come from the positionality of the authorship team.

The authorship team worked together to co-create these principles. Following completion of an initial manuscript, all authors were asked to develop three to five focused recommendations based on the literature and arguments laid out in the paper. Recommendations from each author were presented during the meeting and, in acknowledgement of power dynamics within an authorship team of varying levels of seniority, all authors also had the option to submit their contributions via a google form. Each recommendation presented and submitted via the form was given careful consideration during the meeting. Recommendations included in this paper were selected by discussion during the meeting. There was overlap between many proposed points, and these were combined where reasonable.

The structure for presentation of the recommendations was also established collaboratively through a consensus decision to employ the levels of society (individual, community, institution and health system, and structural) as per Devakumar and colleague's model from the *Lancet Series on racism, xenophobia, discrimination, and health* presented in Figure 1.
